# Auditory Domain Sensitivity and Neuroplasticity-Based Targeted Cognitive Training in Autism Spectrum Disorder

**DOI:** 10.3390/jcm12041635

**Published:** 2023-02-18

**Authors:** Angela Tseng, Megan DuBois, Bruno Biagianti, Caroline Brumley, Suma Jacob

**Affiliations:** 1 Department of Psychiatry & Behavioral Sciences, University of Minnesota, Minneapolis, MN 55455, USA; 2 Department of Psychology, University of Milano-Bicocca, 20122 Milan, Italy

**Keywords:** auditory, autism, cognitive training, remote delivery, sensory processing, web-based

## Abstract

Sensory processing, along with the integration of external inputs into stable representations of the environment, is integral to social cognitive functioning; challenges in these processes have been reported in Autism Spectrum Disorder (ASD) since the earliest descriptions of autism. Recently, neuroplasticity-based targeted cognitive training (TCT) has shown promise as an approach to improve functional impairments in clinical patients. However, few computerized and adaptive brain-based programs have been trialed in ASD. For individuals with sensory processing sensitivities (SPS), the inclusion of some auditory components in TCT protocols may be aversive. Thus, with the goal of developing a web-based, remotely accessible intervention that incorporates SPS concerns in the auditory domain, we assessed auditory SPS in autistic adolescents and young adults (N = 25) who started a novel, computerized auditory-based TCT program designed to improve working memory and information processing speed and accuracy. We found within-subject gains across the training program and between pre/post-intervention assessments. We also identified auditory, clinical, and cognitive characteristics that are associated with TCT outcomes and program engagement. These initial findings may be used to inform therapeutic decisions about which individuals would more likely engage in and benefit from an auditory-based, computerized TCT program.

## 1. Introduction

Clinical reports of sensory sensitivities in Autism Spectrum Disorder (ASD) often refer to autistic persons’ heightened awareness of minute details that are not perceived or noticed by most. Patients may share, for example, that they can distinguish between roads while riding in a vehicle just by listening to fluctuations in the sound of their tires on different surfaces, are irritated by one person’s gum-chewing in a house rife with ambient noises, or that they dislike fluorescent bulbs because they can see the lights flashing on/off above the critical flicker frequency of 50 Hz. ASD is a complex neurodevelopmental disorder (NDD) characterized by persistent deficits in communication and interaction and restrictive, repetitive patterns in behavior, interests, and activities [1]. Although sensory processing sensitivities (SPS) have been reported in ASD since the earliest descriptions of autism [2], only in the last decade has “hyper- or hypo-reactivity to sensory input or unusual interests in sensory aspects of the environment” been added to standard diagnostic criteria [1,3]. While myriad therapeutic approaches have been trialed to remediate social functioning challenges in ASD, few have incorporated SPS as a key factor in intervention success or failure [4,5]. Yet, recent estimates of sensory symptom prevalence in ASD range from 69% to as much as 93% [6,7]. Moreover, SPS correlates highly with levels of autistic traits in the general population [8].

Sensory processing is crucial for forming reliable environmental precepts upon which other cognitive and adaptive abilities depend. Perturbations in this process may originate from ASD-related differences in low-level processing in sensory-dedicated regions in the brain [9,10] and continue to have ramifications across the lifespan. In effect, difficulty sensing, processing, and integrating the dynamic elements of social stimuli into a stable representation may be etiological in ASD, associating strongly with impaired community functioning, limited independent living capabilities, and reduced quality of life [11]. Critically, in the auditory domain, atypical processing early in life may impact later downstream neural mechanisms that contribute to the development of speech and communication skills key to social cognition. From this perspective, presentations of ASD may be better understood through atypical sensory processing characteristics [12] and how individuals with varying cognitive-behavioral profiles navigate and experience their environments.

In recent years, the prevalence of ASD has reached an estimated 1-in-44 in the United States [13], and the lifetime cost of care has been associated with approximately $3.6 million [14]. As such, the development of effective and accessible interventions to address challenges experienced by autistic individuals is urgently needed. Frequently, treatment plans not only need to address core diagnostic symptoms, but also a variety of co-occurring developmental, psychiatric, neurologic, or medical diagnoses that further impact daily functioning and quality of life [15]. Targeted cognitive training (TCT) based on the mechanism of neuroplasticity has emerged as a promising approach to improve functional impairments in multiple pathologies, e.g., general cognitive deficits in chronic schizophrenia [16,17,18] and in children and adolescents with attention-deficit/hyperactivity disorder (ADHD) [19,20]. Neuroplasticity refers to the inherently dynamic biological capacity of the central nervous system (CNS) to undergo maturation, change structurally and functionally via synaptic pruning and myelination in response to experience, and adapt following injury [21]. The first “window of opportunity” for neuroplasticity during early childhood has been well established as a critical period to influence the developing brain, as key neuronal connections (e.g., sensory, motor, language) are constructed and consolidated [22,23]. These processes begin to reach adult levels in mid-adolescence [22,24], a period of heightened experience-dependent learning considered a second window for neuroplasticity when dynamic interactions among physical, sexual, and brain development coincide with increased independence and exposure to novel contexts [25,26,27,28,29]. During adolescence, neural networks underpinning affective information processing functionally mature [30,31], leading to adaptive changes in how adolescents experience and regulate emotions in response to social cues, as well as how they understand the social world and others’ mental states [32,33]. However, exposure to complex interpersonal situations and peer networks can be challenging for autistic youth; avoidance of social interactions may preclude meaningful social experiences and impede social skills development [34]. Longitudinal studies of adults with ASD and without an intellectual disability have shown consistent and persistent deficits across cognitive, social, and vocational domains [35]. Moreover, the cognitive and social skill deficits that are core features of ASD have been identified as major challenges to employment success for these adults highlighting the critical need for evidence-based interventions [36] as autistic adolescents transition into adulthood. Thus, capitalizing on the putative second window of opportunity for neuroplasticity [27,28,29], we recruited autistic adolescents and young adults to participate in a computerized auditory-based TCT program designed to leverage brain plasticity.

To date, a number of studies have examined the efficacy and utility of computer-based TCT programs in clinical and developmental samples [37,38,39]. Although findings have been inconsistent, e.g., [39,40,41], both near-transfer (improvement of the trained function) and far-transfer (improvement of other functions) have been reported [16,42,43,44,45,46,47,48,49]. Yet, few TCT programs have been designed and evaluated for autistic individuals. While the development of remotely administered, web-based interventions confers increased accessibility and a broader reach of TCT for the ASD community [50,51,52], the time commitment required for the successful implementation of each program must be considered when determining treatment suitability for individuals. Protocols using auditory-based TCT have yielded positive results in schizophrenia and ADHD trials [17,18,19,20,53,54]; however, because these interventions employ aurally-presented stimuli, challenges in the auditory domain may impede or reduce the acquisition of cognitive gains for those individuals.

Thus, the goals of our study were as follows: (1) to assess autistic response to a remotely-delivered auditory-based TCT program with consideration for feasibility and efficacy; (2) to evaluate the effects of auditory SPS on intervention engagement and compliance; and (3) to identify participant characteristics at baseline that associate with training outcomes. Our long-term objective is to inform and better tailor interventions for autistic individuals.

## 2. Methods

We recruited individuals with a prior ASD diagnosis (N = 25, 4F, Mean Age = 17.4 ± 4.9 years, IQ ≥ 70) from local clinics and the community to participate in a customized, adaptive, auditory-based TCT program.

Diagnosis was confirmed by our clinical team with autism expertise through obtained medical history and comprehensive review of developmental and evaluation records. Participants with co-morbid diagnoses of ASD-related neurodevelopmental disorders (ADHD, obsessive–compulsive disorder—OCD) were included (see Table 1); these comorbidities commonly occur with ASD and were assessed independently. Exclusion criteria included inability to consent, limited English proficiency, and significant medical, cognitive, or behavioral conditions that precluded testing. Participants and parents/guardians completed informed consent and then in-clinic assessment sessions before and after web-delivered, computerized training.

At the pre-intervention assessment session, we collected parent-report and/or self-report measures (when developmentally appropriate), including demographic, family medical history, the *Pediatric Quality of Life Inventory* (PedsQL) [55], the *Social Responsiveness Scale, 2nd Edition* (SRS-2) [56], the *Repetitive Behavior Scale—Revised* (RBS-R) [57], *Child and Adolescent Symptom Inventory* (CASI-4R) [58]. To assess cognitive functioning, participants completed the tablet-administered brief assessment of cognition (BAC App) [59] and the Minnesota executive functioning scale (MEFS) [60] with researchers. We evaluated auditory processing using the filtered words (FW) and the competing words (CW) subtests from the SCAN Test for auditory processing disorders [61]. The FW subtest assesses the ability to decipher human speech from background sounds by presenting single monosyllabic words that have been low-pass filtered at 750 Hz to reduce intelligibility. The CW subtest is a dichotic listening task that presents two monosyllabic words presented simultaneously, one word to each ear, and identifies developmentally delayed or damaged central auditory pathways. The test-taker is asked to repeat the target word presented via headphones, which may be directed to either the left or the right ear. We opted to analyze raw scores from measures because standardized scores based on U.S. population-based norms may not be appropriate for an autistic sample. Participants who completed the training program were then asked to complete a post-intervention assessment session, which included the same measures as those administered at baseline.

The neuroplasticity-based auditory-based TCT program comprised multi-level, computerized exercises designed to enhance the speed and accuracy of auditory information processing while engaging neuromodulatory systems involved in attention and reward [53]. Participants engaged over the course of 12–16 weeks, during which time they were asked to complete 640 exercise levels or up to 1440 min of training; the intervention was accessed by each participant via the web-based platform on a computer/tablet device with headphones. The program consisted of four adaptive training exercises that are commercially available from Posit Science Corporation (San Francisco, CA, USA); study investigators paid for participant access to the BrainHQ platform. Prior studies with clinical samples (e.g., schizophrenia, ADHD) have reported cognitive gains from engaging in the selected exercises [19,20,46,53,62].

For the present investigation, we requested a customized research training module from Posit Science that only included the following auditory-based “games” (demonstrations available at https://www.brainhq.com/why-brainhq/about-the-brainhq-exercises/, accessed on 12 December 2022):

(a) Auditory Sweeps (called “Sound Sweeps” in the platform) target auditory processing speed (units = msec). Two successive frequency-modulated tone sweeps were presented, and participants indicated whether the frequency increased or decreased within each tone;
(b) Sound Discrimination (“Fine Tuning”) targets auditory perception and processing speed (units = msec). Participants indicated which one of two confusable syllables was presented;
(c) Syllable Ordering (“Syllable Stacks”) targets auditory memory (units = # items). Participants reported the order of presented syllables in a serial memory span task;
(d) Auditory Spatial Match (“Memory Grid”) targets auditory memory (units = # items). Participants matched identical cards representing syllables.

Each block level consisted of 20–50 adaptive trials, and the difficulty level of each trial depended on an individual’s performance in previous trials. Completion of each level was based on user performance; once exercise-specific algorithms detected a lack of additional improvements, the block terminated, and a new block from the same exercise was presented. TCT exercises adjusted difficulty level to user performance to maintain an approximately 80% rate of correct responses; trials with correct responses were rewarded with points and animations. Generally, exercises progressed in a defined order of difficulty, moving from more simple levels (e.g., easy-to-discriminate stimulus types, fewer response options) to more complex levels (e.g., greater rule complexity, greater similarity between stimuli). All performance metrics were recorded throughout the training course. To improve compliance and retention, research staff scheduled weekly video/phone chat check-ins with each individual participant.

### Data Analyses

The overall effects of completing the intervention were examined by conducting repeated-measures analysis of covariance (ANCOVA) tests (IBM SPSS Statistics for Windows, Version 25) on pre-/post-intervention clinical and cognitive measures for participants, covarying for age and sex, who completed all levels of the auditory-based TCT program.

TCT performance was evaluated according to metrics derived by Posit Science Corporation [63]: (1) baseline performance (**avg.orig**) = score reached the first time a participant encountered each exercise; (2) number of blocks/levels (**num**) = direct measure of exposure to a training exercise; (3) peak performance (**avg.best**) = best score reached in a training exercise at any point throughout the intervention; (4) weighted peak performance (**wavg.best**) = weighted average of peak performance that takes into account the number of blocks completed by each user for that specific exercise; (5) delta (**avg.delta**) = difference between subject-specific best and baseline performance within that block, averaged across blocks; (6) weighted delta (**wavg.delta**) = avg.delta divided by the standard deviation of baseline performance for that block across all study participants. The weighted peak performance metric allowed us to consider improvement in speed and accuracy for participants at varying levels of the training curriculum. In addition to serving as a measure of exposure to the TCT program, the number of blocks completed was also considered an indirect measure of participant engagement (i.e., willingness to continue with training exercises).

Two-step cluster (TSC) analyses were applied to identify subgroups of intervention exposure and engagement; the total number of levels completed was input as a continuous variable. TSC analysis employed a log-likelihood distance measure, Akaike’s information criterion (AIC) clustering criterion, and a maximum of 15 clusters. Silhouette coefficients of cohesion and separation, along with membership variables of each cluster solution, were used to assess whether outcome measurements served as accurate indicators of identified subgroups which we designated high-, mid-, and low-engagement groups. ANCOVAs were then used to compare performance metrics by cluster groups.

Post-hoc, exploratory analyses using partial correlations controlling for age and sex (one-tailed significance) were conducted to identify baseline factors that are associated with level of engagement and intervention performance metrics; we posit that these factors may be used to inform therapeutic decisions about which individuals will be more likely to utilize and then benefit from this auditory-based TCT program.

## 3. Results

### 3.1. Overall Effect of Auditory-Based TCT

**Figure 1:**
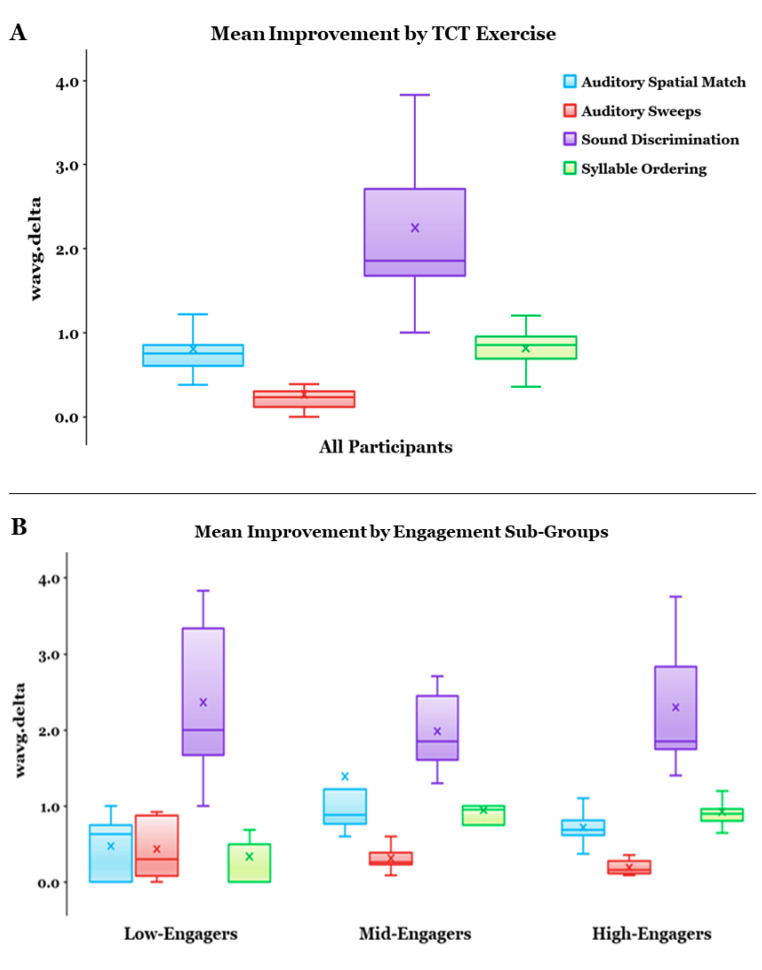
(**A**) Mean weighted average performance change (wavg.delta) for each auditory-based targeted cognitive training (TCT) exercise, calculated by taking the difference between participant-specific best and baseline performance divided by the standard deviation of baseline performance across all study participants. (**B**) wavg.delta for each TSC-Based Engagement Sub-Group suggests that non-completers (Low-/Mid-Engagers) demonstrate improvement on tasks in a pattern similar to completers (High-Engagers). However, data from low-engagers should be interpreted with caution as their limited exposure to the TCT exercises was likely insufficient to effect/measure meaningful change.

Weighted average change metrics were calculated for all participants (N = 25) who started the auditory-based TCT program regardless of whether they completed all levels of training; mean performance change (wavg.delta) on all four exercises reflected improvements from baseline: Sound Discrimination = 2.248, SD = 0.795; Syllable Ordering = 0.812, SD = 0.373; Auditory Sweeps = 0.265, SD = 0.265; Auditory Spatial Match = 0.803, SD = 0.624 (Figure 1A). In summary, 15 of the participants completed all 640 levels of the intervention program, and 10 completed some but not all levels.

**Figure 2:**
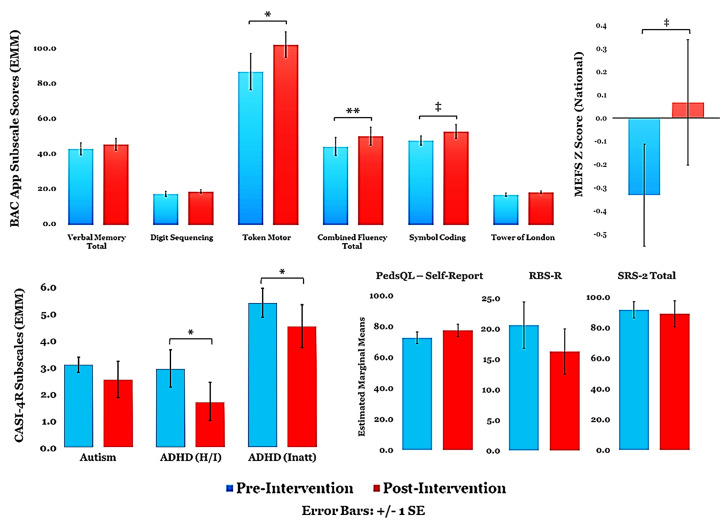
Mean pre-/post-intervention scores on clinical and cognitive assessments. Covariates appearing in the model are evaluated at the following values: Age = 17.04, Sex = 0.17. ** *p* < 0.01; * *p* < 0.05; ‡ *p* < 0.10 (trend).

Due to COVID-19 restrictions, we were unable to collect the entire battery of post-intervention measures for some completers. For the subset of participants who provided pre- and post-intervention data, we found significant within-subject improvements in clinical and cognitive measures (Figure 2): BAC App Token Motor Subscale: F (1,9) = 8.625, *p* = 0.017 η^2^ = 0.489; BAC App Letter Fluency Total: F (1,9) = 5.716, *p* = 0.041; η^2^ = 0.388; BAC App Combined Fluency Total: F (1,9) = 11.044, *p* = 0.009; η^2^ = 0.551; CASI-4R ADHD-Hyperactivity/Impulsivity Subscale: F (1,9) = 6.249, *p* = 0.034; η^2^ = 0.410; and the CASI-4R ADHD-Combined Subscale: F (1,9) = 5.413, *p* = 0.045; η^2^ = 0.376. We found trend-level improvements on the BAC App Symbol Coding Subscale: F (1,9) = 4.801, *p* = 0.056, η^2^ = 0.348, and the MEFS App Total Score (national z-score): F (1,9) = 4.354, *p* = 0.067, η^2^ = 0.356. Changes in the following pre-post-intervention measures did not reach significance: CASI-4R Autism Subscale: F (1,9) = 0.851, *p* = 0.380, η^2^ = 0.086; SRS-2 Total: F (1,8) = 0.159, *p* = 0.701, η^2^ = 0.019; RBS-R Total: F (1,6) = 1.561, *p* = 0.258, η^2^ = 0.206; PedsQL-Self Report: F (1,9) = 2.883, *p* = 0.124, η^2^ = 0.243.

### 3.2. TCT Performance

TSC analysis of the total number of levels completed (num) for all participants yielded a three-cluster solution with a robust silhouette measure of cohesion and separation coefficient (0.9644); we designated these engagement subgroups as High (Mean Levels: 640), Mid (Mean Levels: 322), and Low (Mean Levels: 42.8). Mean weighted average performance change for each TCT exercise for each TSC-Based Engagement Sub-Group suggests that non-completers (Low-/Mid-Engagers) demonstrate improvement on tasks in a pattern similar to completers (High-Engagers) (Figure 1B). However, performance metrics from low-engagers should be interpreted with caution since their level of exposure to the intervention was likely insufficient to effect/measure meaningful change. Nevertheless, TSC analysis yielded the most robust solution when “low-engagers” were retained, suggesting potentially measurable distinctions in the behavioral phenotypes of each cluster.

**Figure 3:**
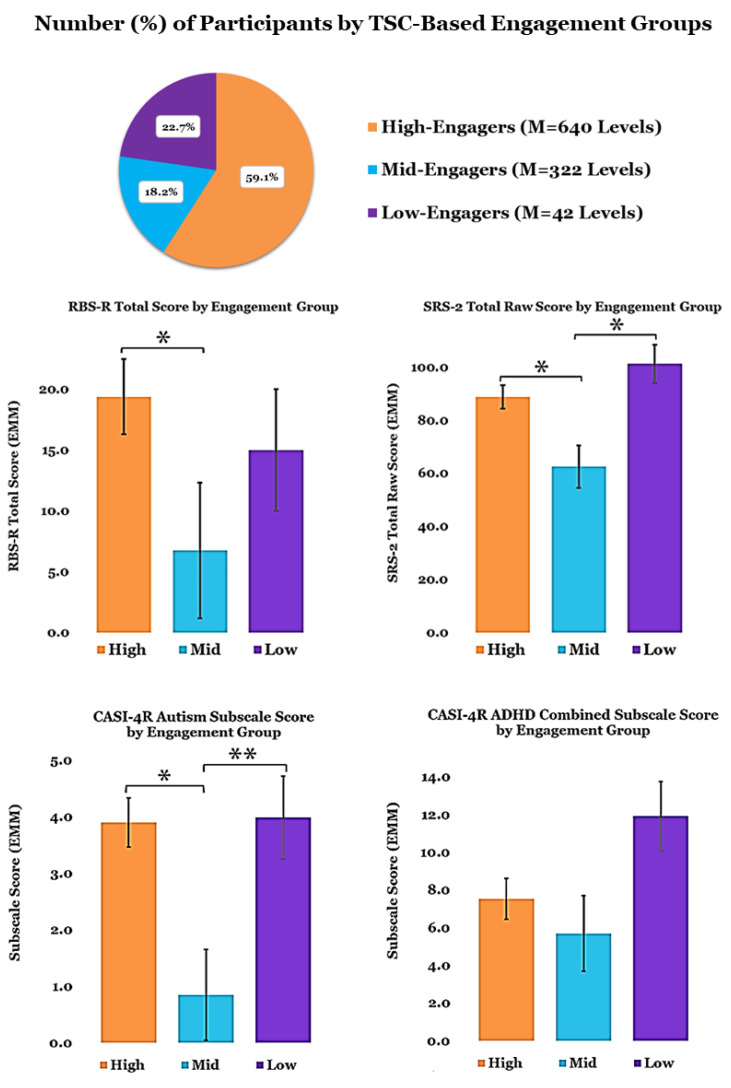
Two-Step Cluster Analysis-Based Engagement Groups (Mean Targeted Cognitive Training Levels Completed: High = 640; Mid = 322; Low = 42.8).; Baseline clinical assessment scores by Engagement Sub-Grouping. Covariates appearing in the model are evaluated at the following values: Age = 17.0, Sex = 0.18. Error Bars = ±1 SE; ** *p* < 0.01, * *p* < 0.05.

Participant characteristics (age, sex, diagnosis) did not differ significantly across the three subgroups. However, scores on clinical measures collected at baseline showed differentiation by TSC solution: SRS-2 Total (F (4,17) = 4.755, *p* = 0.009, η^2^ = 0.528); RBS-R Total (F (4,17) = 4.773, *p* = 0.009, η^2^ = 0.527); CASI-4R ADHD Combined (F (4,17) = 3.269, *p* = 0.037; η^2^ = 0.435, (pairwise comparisons n.s.)); CASI-4R Autism (F (4,17) = 3.004, *p* = 0.048, η^2^ = 0.414). Pairwise comparisons with Bonferroni corrections revealed a significant mean difference between High- and Mid-Engagers on the SRS-2 Total Score (*p* = 0.050) and RBS-R Total Score (*p* = 0.033) and between Mid- and Low-Engagers (*p* = 0.003) on the SRS-2 Total Score. A trend-level difference between High- and Low-Engagers was also found (*p* = 0.077) for the SRS-2 Total Score. High- and Mid-Engagers (*p* = 0.011) and Mid- and Low-Engagers (*p* = 0.035) differed significantly on the CASI-4R Autism Subscale; pairwise comparisons were not significant for between engagement groups on the CASI-4R ADHD Combined Subscale (Figure 3).

**Figure 4:**
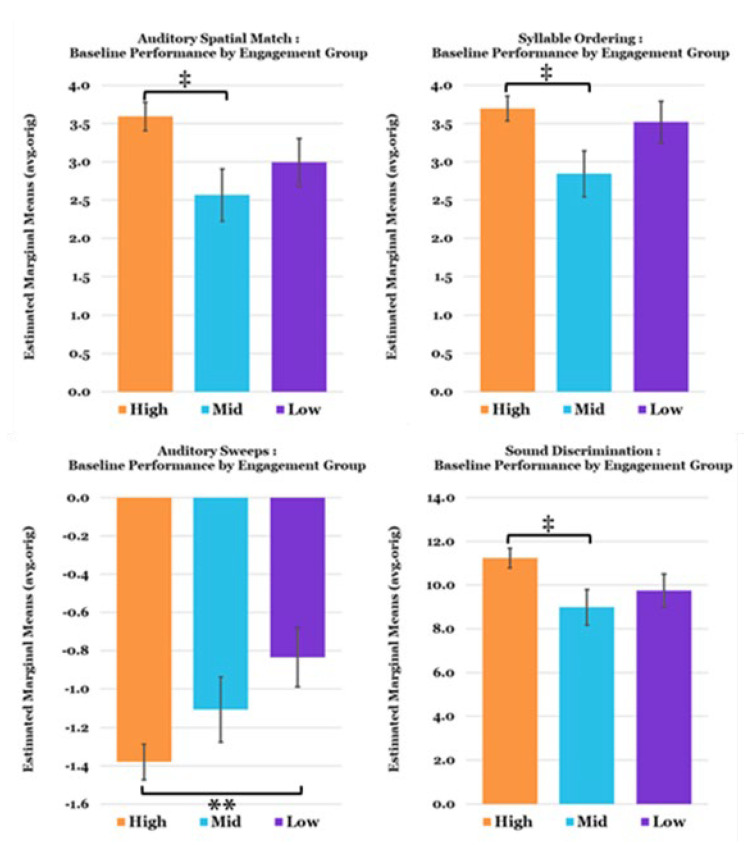
Mean baseline TCT exercise performance by Engagement Group (Mean Levels completed: High = 640; Mid = 322; Low = 42.8). Note that negative values on the performance metric for Auditory Sweeps exercise indicate better performance (faster auditory processing speed). Covariates appearing in the model are evaluated at the following values: Age = 17.0, Sex = 0.18. Error Bars = ±1 SE; ** *p* < 0.01, ‡ *p* < 0.10 (trend).

At baseline, a significant difference in task performance (avg.orig) was found between engagement groups for Auditory Sweeps (F (4,17) = 4.1094, *p* = 0.017, η^2^ = 0.491) and Sound Discrimination (F (4,17) = 3.528, *p* = 0.029, η^2^ = 0.454), as well as a trend level difference for Auditory Spatial Match (F (4,17) = 2.574, *p* = 0.075, η^2^ = 0.377). Pairwise comparisons with Bonferroni corrections revealed a significant mean difference between High- and Low-Engagers on Auditory Sweeps (*p* = 0.024), such that High-Engagers performed better at baseline. Trend level differences were also found between High- and Mid-Engagers on the Auditory Spatial Match (*p* = 0.052), Syllable Ordering (*p* = 0.068), and Sound Discrimination (*p* = 0.078) exercises indicating that High-Engagers performed more accurately than Mid-Engagers (Figure 4).

**Figure 5:**
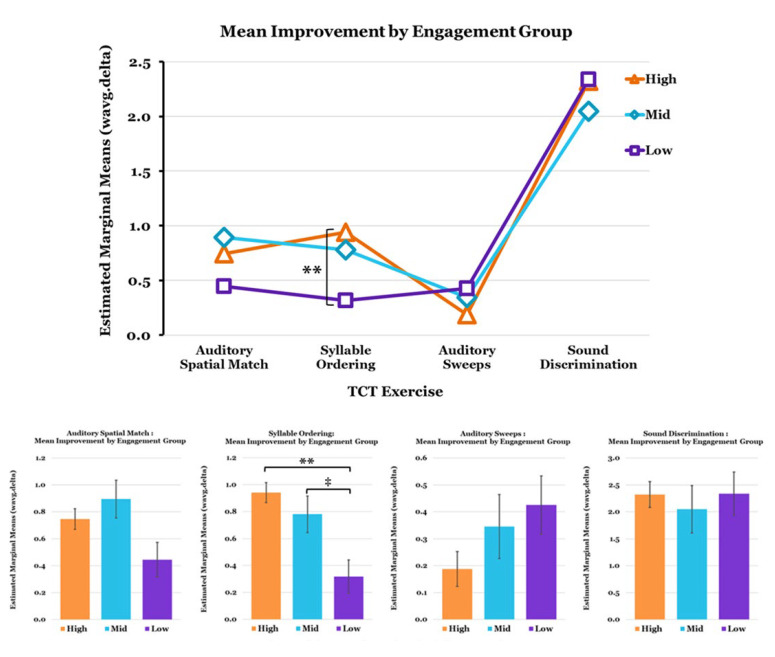
Mean Improvement on TCT Exercises (wavg.delta) by TSC Analysis-Based Engagement Sub-Groups. Covariates appearing in the model are evaluated at the following values: Age = 17.0, Sex = 0.18. Error Bars = ±1 SE; ** *p* < 0.01, ‡ *p* < 0.10 (trend).

A significant main effect for the engagement group on mean improvement in performance (wavg.delta) was found for the Syllable Ordering exercise (F (4,17) = 4.728, *p* = 0.010 η^2^ = 0.527). Pairwise comparisons with Bonferroni adjustments revealed a significant difference between High- and Low-Engagers (*p* = 0.002) and a trend level difference (*p* = 0.078) between Mid- and Low-Engagers (Figure 5).

Partial correlation analysis revealed significant relationships between the number of TCT levels completed and baseline performance on Auditory Spatial Match (*r* = 0.458, *p* = 0.021), Auditory Sweeps (*r* = −0.613, *p* = 0.002), and Sound Discrimination (*r* = 0.433, *p* = 0.028). Significant partial correlations were also found for TCT levels completed and mean improvement on Syllable Ordering (*r* = 0.727, *p =* 0.000) and Auditory Sweeps (*r* = −0.448, *p* = 0.024); a trend level correlation was found for TCT levels completed and mean improvement on the Auditory Spatial Match exercise (*r* = 0.332, *p* = 0.076). Partial correlations for TCT performance metrics are reported in Table 2.

### 3.3. Relationships between Metrics and Predictors of TCT Performance

**Figure 6:**
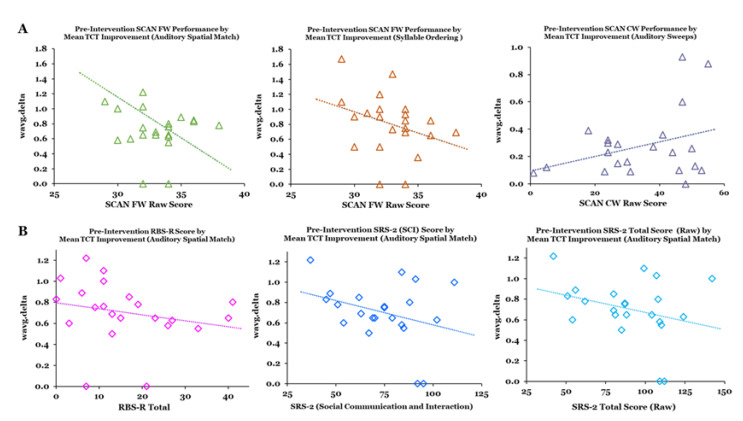
(**A**) Significant (*p* < 0.05) partial correlations between mean TCT exercise improvement (wavg.delta) and Pre-Intervention SCAN Performance Scores. (**B**) Trend-level partial correlations between mean TCT exercise improvement (wavg.delta) and Pre-Intervention clinical assessment scores.

Controlling for age and sex, our analysis of the relationships between baseline SCAN FW raw scores and mean improvement in performance yielded significant partial correlations for the Auditory Spatial Match (*r* = −0.449, *p* = 0.024) and Syllable Order exercises (*r* = −0.397, *p* = 0.041). A significant partial correlation was also found for the relationship between baseline SCAN CW raw scores and mean improvement in performance on Auditory Sweeps (*r* = 384, *p* = 0.047) (Figure 6A). Trend level correlations were found for the relationships between mean improvement on the Auditory Spatial Match exercise and clinical measures at baseline, including the SRS-2 Social Communication and Interaction (SCI) factor (*r* = −0.370, *p* = 0.054) and the RBS-R Total Score (*r* = −0.345, *p* = 0.068) (Figure 6B).

## 4. Discussion

The present study is among the first to examine neuroplasticity-based TCT in ASD; our initial findings support the use of web-based, adaptive, auditory-based TCT to improve outcomes for autistic individuals. Data indicate significant within-subject gains in performance on all four exercises in the adaptive training program. Moreover, participants who completed the program showed improvements in pre- and post-intervention measures of clinical and cognitive function. These data also demonstrate the feasibility of implementing an intense 12–16 week long, remotely-delivered TCT program in autistic adolescents and young adults.

Given that sufficient exposure to the training exercises is necessary to effect meaningful change in target skill domains, a key study design concern is participant engagement. While we could not control the myriad extrinsic circumstances (e.g., illness, school/workload, internet reliability) that may affect whether or not individuals complete all study procedures, we aimed to reduce intrinsic barriers by identifying measurable endophenotypic factors associated with program compliance and outcomes. These features may be useful to consider before implementing a time-consuming intervention schedule; screening at baseline may be used to predict therapeutic goodness of fit for patients, reducing ‘drop-out’ by identifying and enrolling those who would more likely engage in and gain from a long-term, self-motivated TCT program. Accordingly, individuals who are less likely to benefit from this form of intervention may be referred to treatments that are more compatible. Interestingly, the mid-engagement group evidenced significantly fewer symptoms (i.e., lower scores) than low- and high-engagers on the RBS-R, SRS-2, and CASI-4R measures at baseline. While further study is required, we conjecture that attrition from higher-functioning individuals who enrolled in the study may be related to loss of interest in the protracted, repetitive program despite the adaptive nature of the exercises.

Our post-hoc analyses evaluating pre-intervention measures of auditory sensitivity and processing show promise as predictors of therapeutic efficacy. Raw scores on the SCAN FW subtest were significantly correlated with engagement and performance change metrics such that participants with more auditory processing difficulties (lower SCAN FW scores) showed greater mean improvement on the Auditory Spatial Match and Syllable Ordering exercise. Similarly, raw scores on the SCAN CW subtest were significantly correlated with weighted average change on the Auditory Sweeps exercise, indicating that lower SCAN CW scores were associated with greater improvement in performance. These pre-intervention SCAN findings align with baseline performance data on Auditory Sweeps, a training task designed to target “bottom-up” auditory processes [46], such that pre-TCT (first exposure) performance correlated significantly with mean improvement on multiple TCT exercises.

The presented findings data underscore the need to shift towards precision treatments that take individual endophenotypes into consideration when developing a therapeutic regimen. Heterogeneity within the ASD diagnosis precludes the efficacy of generalized approaches, yet, the burden of extensive, personalized characterization and treatment can be prohibitive. The present study provides a promising approach to streamlining screening for auditory SPS with objective task-based measures for auditory SPS. While we administered the SCAN FW and CW subscales during in-person assessments, remote administration is also feasible with video-chat technology and headphones. Similarly, the web-based auditory-based TCT program allows for fast electronic data upload and scoring of baseline performance. These brief, remotely-delivered measures allow for expedient decisions about whether an individual is more or less likely to benefit from the intervention.

### Limitations and Future Directions

Our study was limited in a number of ways: (1) the time commitment required to complete the TCT program was lengthy for this population (12–16 weeks); consequently, adolescents with academic, extra-curricular, or away-camp activities were less likely to participate in a study requiring sustained engagement. Moreover, our data collection was hindered by mandatory constraints on active research during the COVID-19 pandemic; hence, our sample size was modest. Fortunately, most of our TCT program could be completed online, and our researchers were able to maintain weekly check-ins via phone/video calls in order to provide support as participants progressed in the program. (2) Future studies will need to include autistic participants asked to play non-adaptive games (e.g., comparison control computerized program) as well as non-autistic participants as comparisons to complete the TCT program (e.g., specificity to ASD or other disorders affecting language and social processing). In this initial TCT study, since our objectives were to better understand individual differences across the spectrum of autism so that we might inform future interventions, we chose to focus on within-subject effects. (3) Our study sample includes a high (5:1) male:female ratio; while this imbalance is roughly comparable to the broader ASD population, the lack of female participants precludes generalization to autistic females. (4) While we posited the predictive potential of pre-intervention measures in determining the suitability of auditory-based TCT, our *post-hoc* analyses of the relationships between factors are correlational and should not be over-interpreted without further investigation. (5) Finally, given the remote-delivery design of the study, we did not include direct observations or assessments of adolescent peer interactions. Nevertheless, we do posit that the demonstrated “near-transfer” of cognitive gains on auditory perception, memory, and speed of processing may lead to “far-transfer” in other domains. For example, we hypothesize that faster processing of auditory information may be beneficial for individuals who have difficulty distinguishing temporal cues in conversation. Future study designs will need to include longer-term follow-up of participants as well as direct measures of potential “far-transferred” skills in practice.

## 5. Conclusions

One of the primary objectives of this investigation was to ascertain which baseline measures associate with intervention outcomes in an effort to better predict which individuals might (A) comply with the demands of a program that requires a significant time commitment and willingness to engage and (B) benefit from the program if they are exposed to enough of the intervention. Given that all participants demonstrated some gains in the TCT skills, we suggest that compliance and engagement may be the limiting factor in intervention success. Participants who demonstrated more auditory processing deficits during the pre-intervention assessment (e.g., SCAN) showed greater average improvement over time, and these gains were associated with the number of levels completed. However, poorer baseline TCT program performance also correlated positively with the number of levels completed. These data suggest that those who might benefit more from greater exposure to the TCT program also had the most trouble staying engaged. As such, future studies will need to evaluate whether individuals with low baseline performance scores can gain more from the TCT program if additional support is provided to increase engagement. Notably, we found that our high engagement group (completed all TCT levels) showed symptom levels between mid- and low-engagers on the RBS-R, SRS-2, and CASI-4R measures at baseline. These findings show that individuals with “mid” level symptoms may be a good fit for TCT as they are more likely to complete the program. Overall, these findings suggest that first encounters with the TCT module provide valuable insight into the potential utility of the auditory intervention for each individual.

Neuroplasticity-based, computerized TCT has emerged as a promising approach to improve outcomes in multiple clinical and cognitive domains. The appeal of evidence-based TCT programs is their broad accessibility (remote delivery), cost-effectiveness, and potential for individual tailoring of treatment targets. Program use and outcomes can be monitored remotely, which allows clinicians and researchers to confirm engagement, provide assistance, and adjust training as needed. Our findings are among the first to provide support for the efficacy and feasibility of long-term, auditory-based TCT for autistic adolescents and young adults. We were also able to identify subsets of participants with similar patterns of symptoms and traits who engaged with and responded to the intervention program; further delineation of these clusters may be used to triage individuals into more appropriate therapeutic plans and inform the development of precision medicine approaches.

## Figures and Tables

**Table 1:** Participant Characteristics.

Participants (N = 25)	
Age (years)	
Mean	17.4
SD	5.0
Sex (%)	
Male	84.0
Female	16.0
Race (%)	
American Indian/Alaska Native	4.0
Asian	4.0
Black/African American	8.0
White/Caucasian	84.0
Other	4.0
Neurodevelopmental Diagnoses (%)	
ADHD, ASD	40.0
ASD	44.0
ADHD, ASD, OCD	12.0
ASD, OCD	4.0

**Table 2:** Partial Correlations between TCT Performance Metrics.

	*M*	*SD*		1	2	3	4	5	6	7	8
avg.orig											
1. Auditory Spatial Match	3.27	0.76	*r*								
			*p*								
2. Syllable Ordering	3.50	0.65	*r*	0.659 **							
			*p*	0.001							
3. Auditory Sweeps	−1.21	0.42	*r*	−0.476 **	−0.374 ‡						
			*p*	0.017	0.052						
4. Sound Discrimination	10.49	1.95	*r*	0.500 **	0.700 **	−0.635 **					
			*p*	0.012	0.000	0.001					
wavg.delta											
5. Auditory Spatial Match	0.70	0.29	*r*	−0.006	−0.086	−0.552 **	0.023				
			*p*	0.490	0.360	0.006	0.462				
6. Syllable Ordering	0.77	0.35	*r*	0.171	0.094	−0.667 **	0.193	0.590 **			
			*p*	0.236	0.346	0.001	0.208	0.003			
7. Auditory Sweeps	0.27	0.24	*r*	−0.360 ‡	−0.329 ‡	0.890 **	−0.607 **	−0.612 **	−0.566 **		
			*p*	0.059	0.078	0.000	0.002	0.002	0.005		
8. Sound Discrimination	2.28	0.83	*r*	−0.340 ‡	−0.176	0.268	−0.325 ‡	−0.144	0.189	0.072	
			*p*	0.071	0.229	0.126	0.081	0.273	0.212	0.382	
num											
9. TCT Levels Completed	446.64	255.61	*r*	0.458 *	0.244	−0.613 **	0.433 *	0.332 ‡	0.727 **	−0.448 *	0.037
			*p*	0.021	0.150	0.002	0.028	0.076	0.000	0.024	0.439

** *p* < 0.01; * *p* < 0.05; ‡ *p* < 0.10 (trend).

## Data Availability

Not applicable.
